# Research on Weak Fault Extraction Method for Alleviating the Mode Mixing of LMD

**DOI:** 10.3390/e20050387

**Published:** 2018-05-21

**Authors:** Lin Zhang, Zhijian Wang, Long Quan

**Affiliations:** 1 College of Mechanical Engineering, Taiyuan University of Technology, Taiyuan 030024, China; 2 Key Laboratory of Advanced Transducers and Intelligent Control System, Ministry of Education, Taiyuan 030024, China; 3 College of Mechanical and Power Engineering, The North University of China, Taiyuan 030051, China

**Keywords:** strong noise, local mean decomposition, fault diagnosis, product function

## Abstract

Compared with the strong background noise, the energy entropy of early fault signals of bearings are weak under actual working conditions. Therefore, extracting the bearings’ early fault features has always been a major difficulty in fault diagnosis of rotating machinery. Based on the above problems, the masking method is introduced into the Local Mean Decomposition (LMD) decomposition process, and a weak fault extraction method based on LMD and mask signal (MS) is proposed. Due to the mode mixing of the product function (PF) components decomposed by LMD in the noisy background, it is difficult to distinguish the authenticity of the fault frequency. Therefore, the MS method is introduced to deal with the PF components that are decomposed by the LMD and have strong correlation with the original signal, so as to suppress the modal aliasing phenomenon and extract the fault frequencies. In this paper, the actual fault signal of the rolling bearing is analyzed. By combining the MS method with the LMD method, the fault signal mixed with the noise is processed. The kurtosis value at the fault frequency is increased by eight-fold, and the signal-to-noise ratio (SNR) is increased by 19.1%. The fault signal is successfully extracted by the proposed composite method.

## 1. Introduction

Bearings are crucial parts of rotating machinery, and bearing wear is inevitable [1,2]. The early signs of bearing wear are very weak, and the signals are difficult to find in the strong noise background [3,4,5]. Bearing faults will occur if corresponding measures have not been taken, which can even lead to serious accidents, causing economic losses and personnel casualties, so the early fault signal extraction of bearings has always been highly valued [6,7,8]. The faults of rolling bearings can be mainly summarized as inner ring wear, outer ring wear and rolling body pitting, and periodic pulse signals will be produced when these faults occur. Due to the noise interference, the early fault signal extraction is always the most important and difficult part of fault diagnosis [9,10]. The existing fault diagnosis methods include traditional Fourier transform, wavelet decomposition and empirical mode decomposition (EMD).

Dragomiretskiy et al. proposed the variational modal decomposition (VMD) method in 2014 [11]. VMD converts the decomposition of the signal into a variational constraint problem. A non-recursive solution is used to avoid the mode mixing which exists in traditional decomposition methods such as empirical mode decomposition (EMD). VMD possesses good noise robustness and high computational efficiency. Wang [12] proposed a VMD method based on particle swarm optimization, which uses the minimum mean envelope entropy to optimize the parameters of VMD and achieved good results in the fault diagnosis of rotating machinery. Yang [13] proposed a method which mixes Ensemble Empirical Mode Decomposition (EEMD), sample entropy and Singular Value Decomposition (SVD) in acoustic signal fault diagnosis, where sample entropy and SVD are indicators of periodic and irregular signals respectively. This adaptive method has good robustness and it is suitable for processing non-stationary and non-linear signals. 

In 2005, on the basis of EMD, Smith proposed a time-frequency analysis method call local mean decomposition (LMD), whose essence is to divide the signal by frequency into different product function (PF) components. Each PF component is an envelope signal multiplied by a pure FM signal. By analyzing PF components after the decomposition in the frequency domain, the complete time-frequency distribution can be obtained [14,15,16].

However, in comparison to EMD some disadvantages of LMD are also obvious. Thus, although the mode mixing of LMD has been reduced, the mode mixing is still serious. The mode mixing results in the mixing of time-frequency distribution and the generation of promiscuous PF components, so the required time-frequency information [17,18] can’t be extracted from them.

In order to solve the mode mixing of LMD, many scholars have proposed different methods in succession. Among these methods, Mask Signal (MS) has attracted much attention due to its high computational efficiency and strong post-processing ability, but so far, MS has not been applied to solve the mode mixing issues of LMD [19,20,21].

Based on the above reasons, this paper introduces MS into LMD, and finds that MS has a certain noise reduction ability, the influence of noise in the PF component has been reduced by MS. The frequency energy mean parameter is the basis of the refinement of the fault frequency band [22]. In the analysis of weak simulation signal and the fault signals of the rolling bearings, the mode mixing of LMD has been successfully weakened, and the fault features have been extracted. The validity of the method is verified by calculating the signal-to-noise ratio and kurtosis ratio before and after the signal processing. In theory and practice, a new diagnostic method for fault feature extraction is provided.

## 2. Background and New Method 

### 2.1. LMD Method

For an original signal x(t), the LMD decomposition steps are as follows:

(1) According to all the local extreme point *n_i_*, all the local extreme mean values *m_i_* and the envelope estimate values *a_i_* are obtained.

The local mean function *m*_11_(*t*) and the envelope function *a*_11_(*t*) are obtained by the sliding average method.

(2) From the local mean function of the original signal *x*(*t*), the function *h*_11_(*t*) is obtained, and then it is demodulated to the function *s*_11_(*t*):

(1) $$h_{11}(t)=x(t)-m_{11}(t)$$

(2) $$s_{11}(t)=\frac{h_{11}(t)}{a_{11}(t)}$$

*s*_11_(*t*) is used as a new function to repeat the above steps to get *s*_12_(*t*) until *s*_1*n*_(*t*) is a pure FM function, that is $\underset{n\to \infty }{\lim}a_{1n}(t)=1$.

(3) The envelope signal is obtained:

(3) $$a_{1}(t)=a_{11}(t)a_{12}(t)\cdots a_{1n}(t)=\prod_{p=1}^{n}a_{1p}(t)$$

(4) The first PF component is obtained:

(4) $$PF_{1}(t)=a_{1}(t)s_{1n}(t)$$

(5) After *PF*_1_(*t*) is separated from *x*(*t*) and *u*_1_(*t*) is obtained, *u*_1_(*t*) repeats the above calculation as a new original signal to obtain *u*_2_(*t*) until *u*_q_(*t*) is a monotone function stop iteration:

(5) $$u_{1}(t)=x(t)-PF_{1}(t)$$

(6) The original signal is decomposed into:

(6) $$x(t)=\sum_{i=1}^{q}PF_{i}(t)+u_{q}(t)$$

### 2.2. MS Method

Deering [23] proposed the mask signal method to alleviate the mode mixing of EMD. The specific way is to determine the amplitude and the frequency of *s*(*t*) according to the EMD’s Hilbert envelope amplitude and instantaneous frequency. Good results have been obtained, but less attention has been paid to the mask signal method in alleviating the mode mixing of LMD.

The basic principle of MS is to reduce the accumulated error of smooth processing by adding and subtracting the average, weaken the mode mixing and noise, highlight the peak value near the mean instantaneous frequency. Finding the proper mask signal *s*(*t*) is the key of MS:

(1) Suppose *t* is time, *x*(*t*) is the original signal, *τ* is the integral variable. We perform a Hilbert transform on the original signal *x*(*t*) to get *y*(*t*), and transform it to *z*(*t*):

(7) $$y(t)=\frac{1}{\pi }\int_{-\infty }^{+\infty }\frac{x(t)}{\tau -t}d\tau$$

(8) $$z(t)=x(t)+jy(t)=a_{i}(t)e^{j\phi_{i}(t)}$$

(2) The instantaneous frequency is obtained according to the instantaneous phases of the amplitude function *a_i_*(*t*) and the phase function *φ_i_*(*t*):

(9) $$f_{i}(t)=\frac{1}{2\pi }\omega_{i}(t)=\frac{1}{2\pi }\frac{d\phi_{i}(t)}{dt}$$

Equation (7) can be calculated by the mean of the energy mean method:

(10) $$\bar{f}=\frac{\sum_{i}^{k}a_{1}(i)f_{1}{}^{2}(i)}{\sum_{i}^{k}a_{1}(i)f_{1}(i)}$$

The average amplitude and the average instantaneous frequency are different when MS is used for different signal processing. In face of *x*(*t*), *a*(*t*) is the envelope amplitude of *x*(*t*), *f*_1_(*t*) is the instantaneous frequency of *x*(*t*), so the mask signal is determined as:

(11) $$s(t)=a_{0}\sin(2\pi \bar{f}t)$$

According to the rule of thumb, *a*_0_ often takes 1.6 times of the average amplitude of the signal component.

(3) Create a mask signal *s*(*t*), let:

(12) $$x_{+}(t)=x(t)-s(t)$$

(13) $$x_{-}(t)=x(t)-s(t)$$

(4) The original signal can be obtained from the combination of *x*_+_(*t*) and *x*_−_(*t*):

(14) $$x(t)=\frac{x_{+}(t)+x_{-}(t)}{2}$$

**Figure 1:**
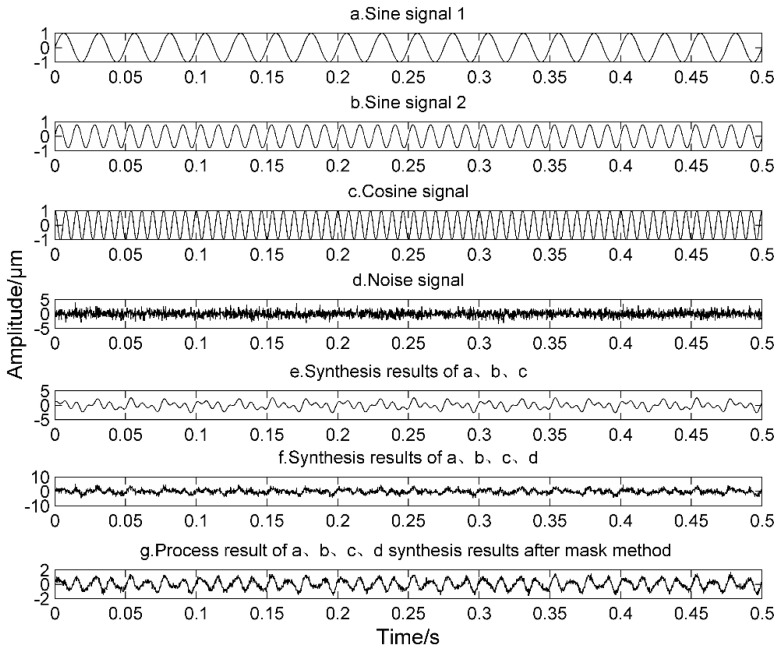
Simulation signal.

The performance of MS is verified by an example of the simulation signal in Equation (15). The simulation signal is shown in Figure 1. The frequencies of a, b and c are 40 Hz, 80 Hz and 130 Hz, respectively:

(15) x(t)=sin(80πt)+0.8sin(160πt)+cos(260πt)+1.5noise(t)

**Figure 2:**
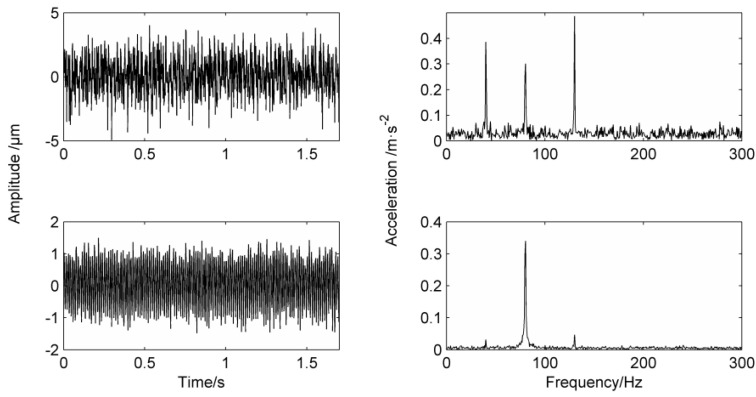
Simulation signal frequency domain of f and g.

As can be seen from the results of e, f, and g in Figure 1, MS has a certain noise reduction capability, and the waveform in the g diagram has also changed. From the comparison results of the frequency domain diagram of f and g in Figure 2, the peak values of the 40 Hz and 130 Hz signals after the masking process are significantly weaker than those of the untreated signal. The peak value of 80 Hz is clearly prominent. This is due to the fact that the relevant parameters of the mask signals of MS are instantaneous amplitude and instantaneous frequencies. The mean instantaneous frequency of the simulation signal is about 83 Hz. After masking, the peaks at 40 Hz and 130 Hz are weakened.

## 3. The Basic Principle of LMD-MS

Nakayama and others put forward a method for improving the performance of the prediction task by mask signal [24], which can be improved appropriately when applied to LMD. Since LMD adopts an envelope based on extreme points, the envelope estimation errors will be amplified after multiple decomposition, and the mode mixing will appear. The MS method takes the mean method after adding and subtracting, which can decrease the accumulated errors resulted from the multiple smooth processing to alleviate the mode mixing and the noise.

Taking the first PF component PF_1_ decomposed by LMD as an example, the decomposed PF_1_ is used as the original signal, and the mask signal is determined, the PF_1_ is decomposed into PF^+^ and PF^−^, redefine PF_1_:

(16) $${\mathrm{PF}}_{1}=\frac{{\mathrm{PF}}^{+}+{\mathrm{PF}}^{-}}{2}$$

When the LMD-MS method deals with a single fault bearing signal, the PF component who has the highest relativity with the original signal can be selected as the target for mask processing.

**Figure 3:**
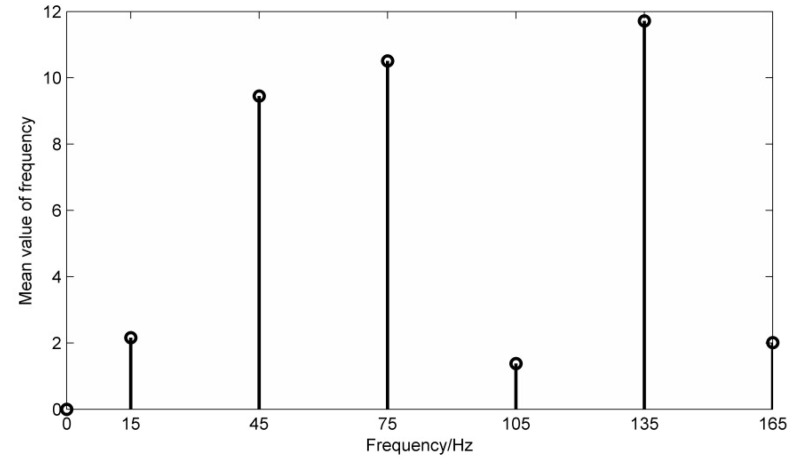
Simulation signal frequency energy mean distribution.

In the fault signal, the fault impact will appear periodically, so in this frequency band, the frequency energy means will be higher than those of other frequency bands. Figure 3 is the frequency energy mean diagram of the simulation signal in Equation (15).

**Figure 4:**
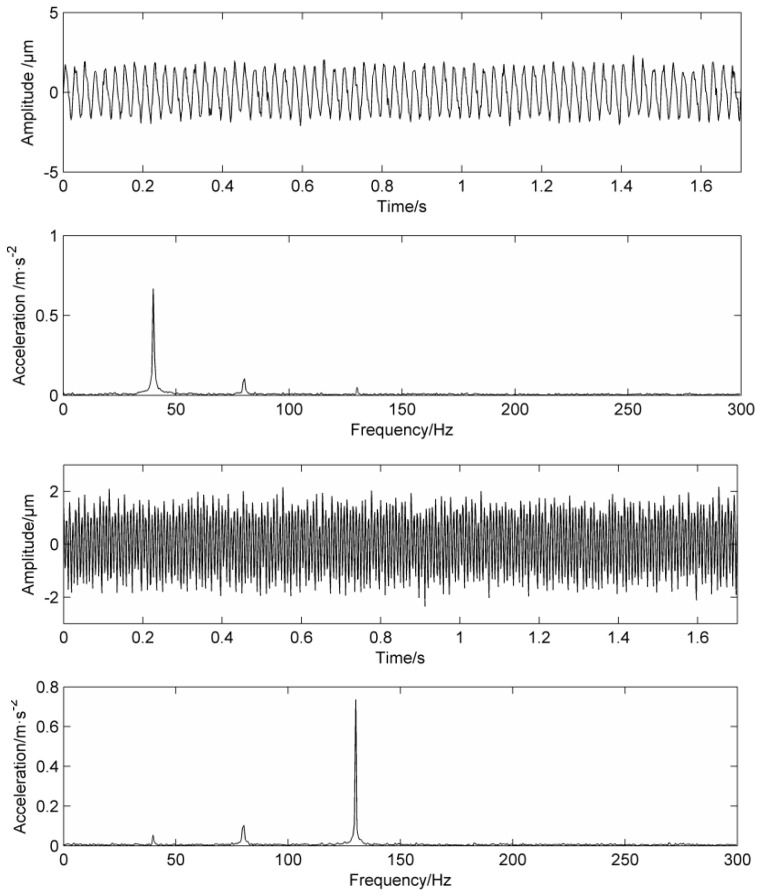
The time-frequency domain of the simulated signal with mean instantaneous frequency of 40 Hz and 130 Hz.

In Figure 3, taking about 15 Hz of each point as the frequency energy mean, it can be obtained that 45 Hz, 75 Hz and 135 Hz are at higher frequency mean energy. When the mean instantaneous frequency of the masking signal is adjusted to 40 Hz and 130 Hz, the results are shown in Figure 4.

**Figure 5:**
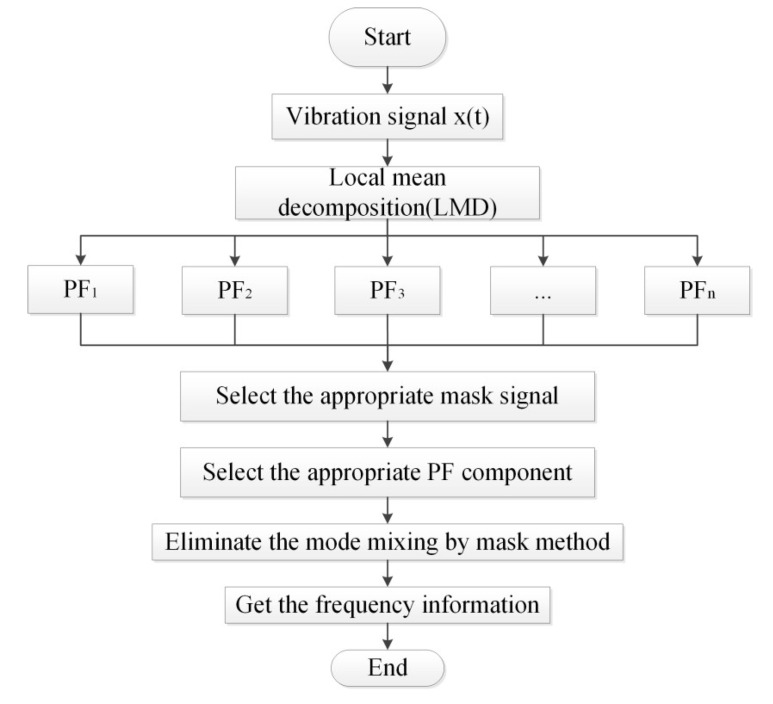
Flow chart of LMD-MS.

When the mean instantaneous frequency is 80 Hz, the time-frequency domain diagram is basically the same as Figure 2, and from the results of Figure 2 and Figure 4, we can see that the selection of the mask signal is the key point. When using the mask method to process the signal, we can choose the frequency with the highest frequency energy as the basis for selecting the mask signal. The flow chart of the fault diagnosis method based on MS and LMD is as follows in Figure 5:

## 4. Simulation Signal Analysis

**Figure 6:**
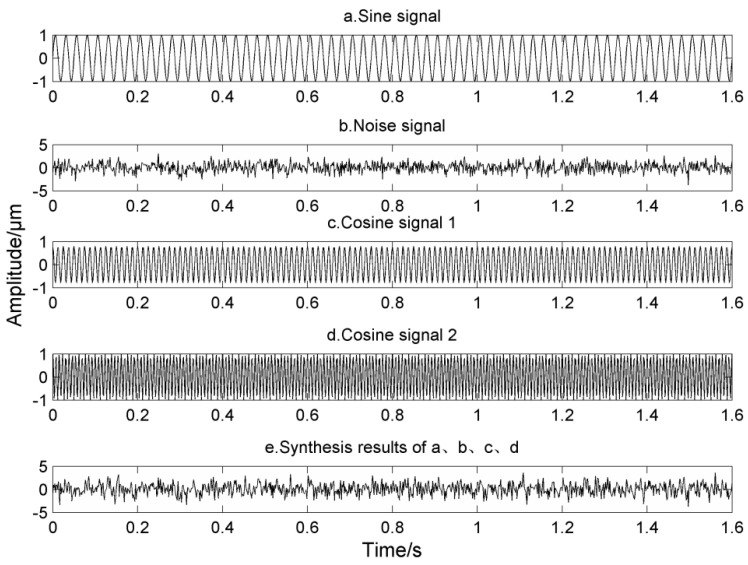
Simulation signal.

In order to verify the mode mixing phenomenon of LMD in noise, the modulated simulation signal in Equation (17) is adopted, the corresponding frequencies are 40 Hz, 120 Hz and 260 Hz respectively, and the corresponding time-domain waveform of simulation signal is shown in Figure 6.

(17) x(t)=[1+0.8cos(240πt)+cos(520πt)]⋅sin(80πt)+noise(t)

In Figure 6, from top to bottom, the signals are a sine signal, the noise signal, two cosine signals and the time domain diagrams of the synthesized simulation signal, where the two cosine functions are modulated by a sine function.

**Figure 7:**
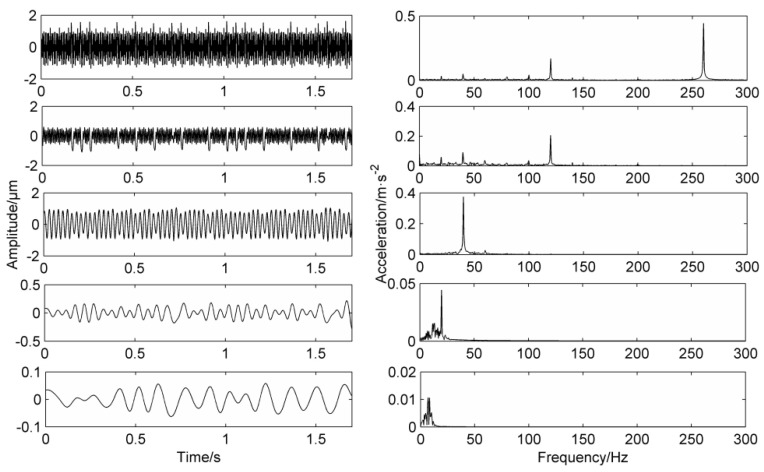
Simulation signal’s decomposition results using LMD.

The frequency domain diagram of the PF component that is directly processed by LMD decomposition without a mask signal method is shown in Figure 7. The first layer is the time-frequency domain of PF_1_, it can be clearly seen from the frequency domain that there are high peaks at 120 Hz, 260 Hz and a lower peak at 40 Hz. The second layer is the time-frequency domain of PF_2_, and there are significant peaks at 40 Hz and 120 Hz. At the same time, there are many false frequencies in the spectrum due to the noise interference. The third layer is the time-frequency domain of PF_3_, the frequency graph only has a higher peak at 40 Hz, the fourth layer and the fifth layer is false components which can be dropped.

From the comparison of the first three layers of the PF components, it can be concluded that 120 Hz and 260 Hz belong to high-frequency components, 40 Hz belongs to low-frequency components, but 120 Hz and 260 Hz appear in the spectrum of PF_1_ at the same time. 40 Hz and 120 Hz appear in the spectrum of PF_2_ at the same time, which proves that mode mixing occurs.

**Figure 8:**
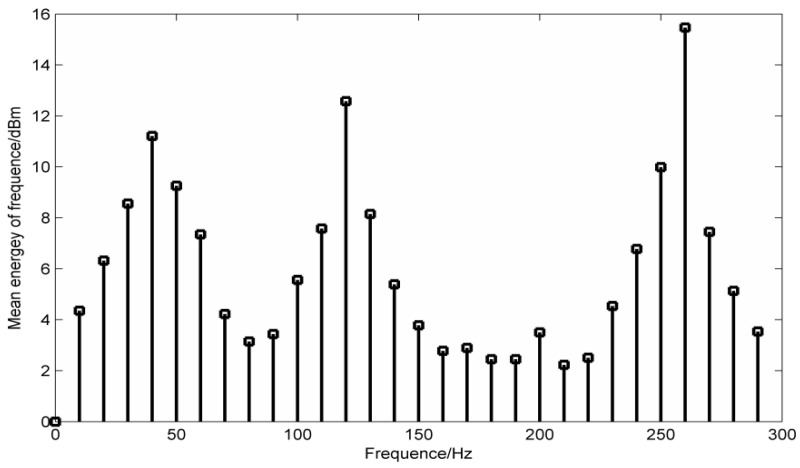
Simulation signal frequency energy mean distribution.

The mean frequency energy parameters of the simulation signal are calculated and the required frequency information is filtered. Regard the frequency value before and after 5 Hz as the frequency energy mean length, which is shown in Figure 8.

The results obtained from Figure 8 show that the mean frequencies of 40 Hz, 120 Hz and 260 Hz are the highest, and the mask signal is selected according to the three frequency values.

When the mean instantaneous frequency of the mask signal is selected as 40 Hz, the corresponding results can be obtained for PF_1_, PF_2_ and PF_3_, but the results obtained for PF_3_ are the best. When the mean instantaneous frequency of the mask signal is 260 Hz, the treatment of PF_1_ is obviously not reasonable. So the choice of processing objects should be adjusted according to the actual situation.

**Figure 9:**
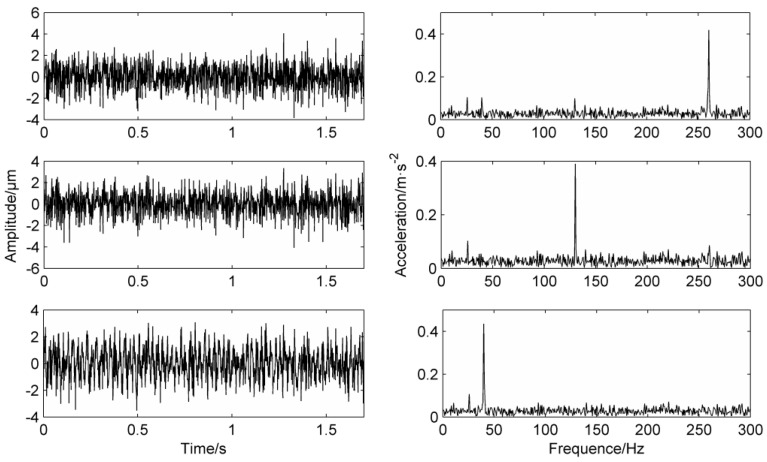
Simulation signal’s decomposition results using LMD-MS.

The selected objects in this paper are as follow: The mean instantaneous frequency of PF_1_ is 40 Hz, the mean instantaneous frequency of PF_2_ is 120 Hz and the mean instantaneous frequency of PF_3_ is 260 Hz. The results are shown in Figure 9.

The first three orders of PF components contain different frequencies. Although there are 40 Hz and 120 Hz frequency components in PF_1_, they have been greatly weakened. Therefore, MS can effectively weaken the mode mixing phenomenon of the simulation signal.

**Figure 10:**
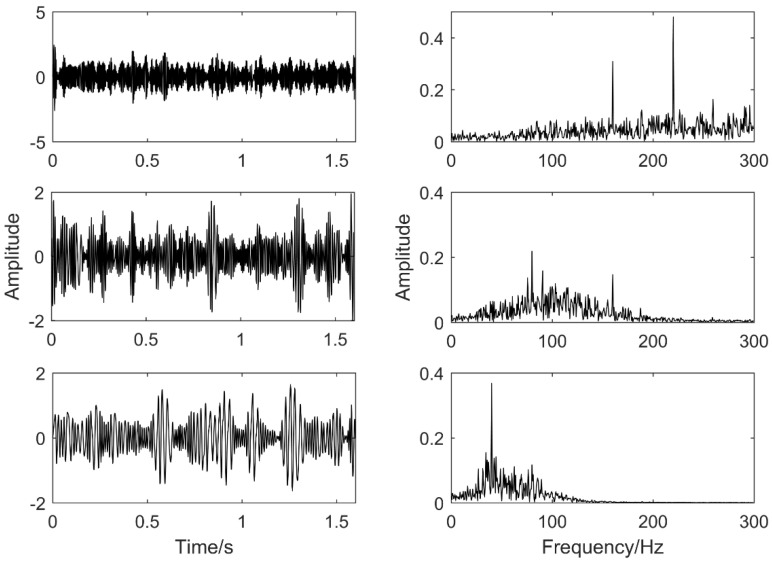
Simulation signal’s decomposition results using EMD.

**Figure 11:**
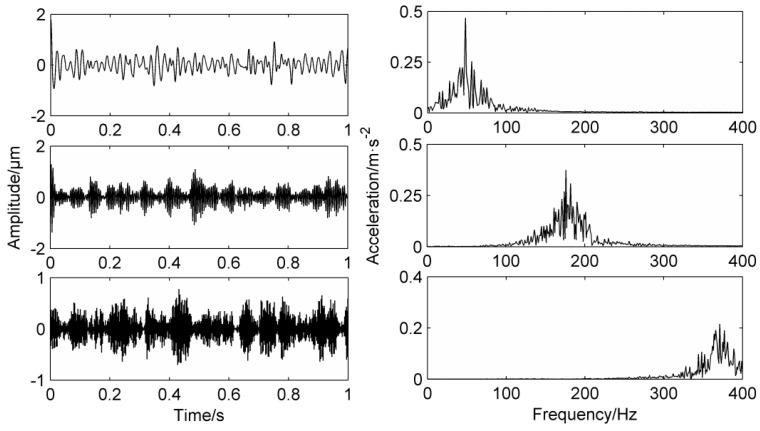
Simulation signal VMD decomposition results.

In order to verify the advantages of the proposed method VMD and EMD are introduced for comparison. Figure 10 is the EMD decomposition results of the simulation signal and Figure 11 is the VMD decomposition results of the simulation signal. From the results of EMD, the frequencies of the decomposed component are 40 Hz, 80 Hz and 160 Hz and 225 Hz. The mode mixing of the EMD decomposition results is very serious and the three components can be obtained. Because the results of VMD are affected by the layer number of its decomposition, the results of decomposition are easily distorted. The simulation signal is composed of 40 Hz, 120 Hz and 260 Hz. From the results of VMD, the frequencies of the decomposed component are 54 Hz, 130 Hz and 335 Hz. Compared with the LMD-MS method proposed in this paper, it is obvious that the decomposition results in the paper are more accurate, which proves that the method proposed in this paper has strong advantages compared with other decomposition methods.

## 5. LMD-MS Rolling Bearing Fault Signal Analysis

**Figure 12:**
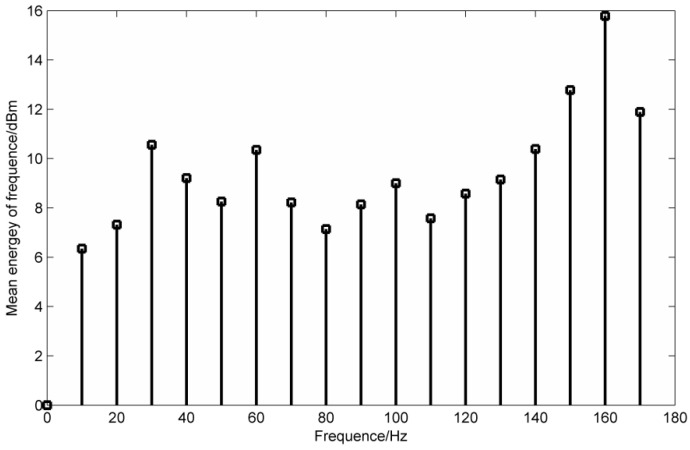
Mean energy distribution of the original signal frequency.

**Figure 13:**
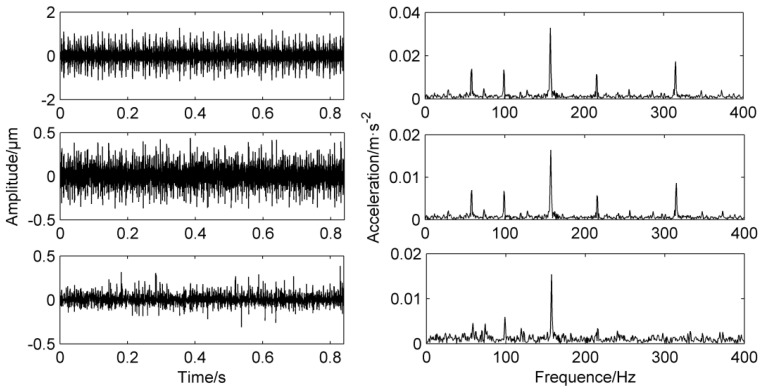
Original fault signal’s decomposition results using LMD and envelope analysis.

In this paper, adopting Case Western Reserve University’s fault bearing data for analysis [25], the speed is 1750 r/min, the sampling frequency is 12,000 Hz, and the basic frequency of the rotating shaft is 29.1 Hz, the calculated inner ring fault frequency is about 157.9 Hz, this paper selects 10,240 points of the data for analysis and research. Calculate the mean frequency energy’s mean value of the original signal and filter the required frequency information. Take the frequency values before and after 5 Hz as the frequency energy mean length are shown in Figure 12. Figure 13 shows the time domain waveforms and envelope analysis results of the LMD decomposition of the fault signal. Take the first three layers PFs that are strongly related to the original signal.

In Figure 12, the mean values of the frequency energy at 29 Hz and 58 Hz are higher, and the mean value of the frequency energy at 160 Hz is the highest, thus the mask signal has been determined.

**Figure 14:**
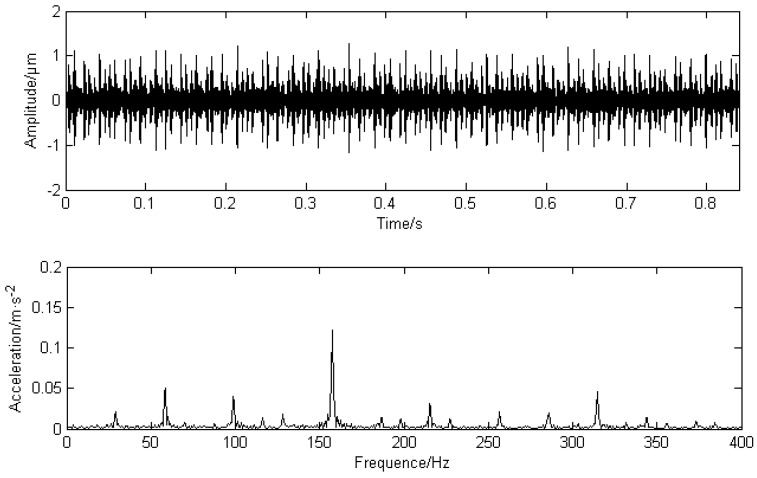
PF1 time-domain diagram and envelope analysis using MS.

In the figure, there are higher peaks at the inner ring fault frequency of 158.3 Hz and its double frequency of 316.6 Hz. Due to the fake frequency modulated by the signal during transmission process, there are higher peaks at the frequency of 29.1 Hz and the double frequency of 58.2 Hz, while 98 Hz and 210 Hz have higher peaks. The results obtained by PF_2_ and PF_3_ are similar to PF_1_, which proves that the mode mixing phenomenon has occurred. PF1 is processed by the mask method and the result is shown in Figure 14. Obviously, the 29.1 Hz and the double frequency of 58.2 Hz are obviously weakened, which weakens the mode aliasing of LMD.

From the comparison of the envelope analysis of the PF_1_ component after the mask processing, it can be seen that the peaks at 58 Hz and 100 Hz are greatly weakened, and higher peaks appear only at the inner fault frequency of 158.1 Hz and the double frequency of 316.2 Hz. At the same time, the signal amplitude is reduced, which proves that MS has a certain noise reduction capability. After calculation, the correlation coefficient of PF_1_ is the highest, so PF_1_ is selected as the research object. In practical application, the component with the highest correlation coefficient can be selected as the research object.

**Figure 15:**
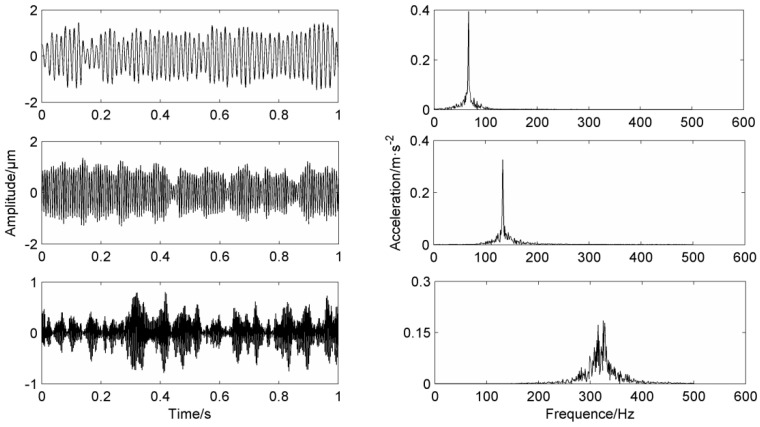
VMD decomposition of the measured signal.

Similar to the simulation signal, VMD is selected as the control method, and the number of selected decomposition layers is 3. The three components of VMD decomposition are shown in Figure 15, and the peak frequencies are at 50 Hz, 120 Hz and 355 Hz respectively. It is obvious that the frequency 157.9 Hz of the inner ring has not been decomposed, which is due to the nature of VMD itself. Therefore, it is proved that the method used in this paper is superior to other fault diagnosis methods.

Optimization degree of LMD-MS is studied from the kurtosis. The kurtosis values of PF_1_, PF_2_ and PF_3_ increased from 3.6, 3.3 and 3.2 to 28.4, 27.6 and 26.8, an increase of nearly eight times.

According to the signal-to-noise ratio formula:

(18) $$SNR=10\mathrm{lg}\frac{2\left|X(k_{0})\right|}{\sum_{k=0}^{L-1}{\left|X(k)\right|}^{2}-2{\left|X(k_{0})\right|}^{2}}$$

The *S*, *N*, and *L* are respectively the signal energy, the noise energy, and the signal length, *X*(*k*) and *X*(*k*_0_) are the peak at *k* and the peak of the characteristic frequency in the spectrum. The signal-to-noise ratio of the signal at 157.9 Hz has increased by 19.1% before and after the use of the mask method.

## 6. Conclusions

(1) MS has a strong inhibitory effect on the mode mixing under strong noise background, and experiments show that MS has a certain ability to the noise reduction.
(2) LMD has a strong ability to decompose and analyze fault signals, but it will distort in strong noise background, and cause mode mixing and so on. It is difficult to get effective fault information.
(3) In this paper, MS is introduced into LMD, and a LMD-MS method which combines the mask signal method with LMD has been proposed. The signal is decomposed by LMD, and then the PF components are processed by MS to reduce the noise and alleviate the mode mixing. The simulation signal is used to verify the feasibility of the proposed method. The signal-to-noise ratio of the actual signal is increased by 19.1%, and the weak fault features of the bearing are extracted successfully. It provides a new research idea for the weak fault feature extraction.

## References

[1] Wang Z.J., Wang J.Y. (2017). Weak Fault Diagnosis of Wind Turbine Gearboxes Based on MED-LMD. Entropy.

[2] Wang Z.J., Wang J.Y. (2018). A novel method for multi-fault feature extraction of a gear box under Strong background noise. Entropy.

[3] Chen G., Hao T.F., Wang H.F., Zhao B., Wang J., Cheng X.Y. (2014). Sensitivity Analysis and Experimental Research on Ball Bearing Early Fault Diagnosis Based on Testing Signal from Casing. J. Dyn. Syst. Meas. Cont..

[4] Tian Y., Ma J., Lu C., Wang Z. (2015). Rolling bearing fault diagnosis under variable conditions using LMD-SVD and extreme learning machine. Mech. Mach. Theory.

[5] Wang Z., Han Z., Gu F., Gu J.X., Ning S. (2015). A novel procedure for diagnosing multiple faults in rotating machinery. ISA Trans..

[6] Li Z., Chen J., Zi Y., Pan J. (2017). Independence-oriented VMD to identify fault feature for wheel set bearing fault diagnosis of high speed locomotive. Mech. Syst. Signal Proc..

[7] Yuan J., Ji F., Gao Y., Zhu J., Wei C., Zhou Y. (2018). Integrated ensemble noise-reconstructed empirical mode decomposition for mechanical fault detection. Mech. Syst. Signal Proc..

[8] Park C.S., Choi Y.C., Kim Y.H. (2013). Early fault detection in automotive ball bearings using the minimum variance cepstrum. Mech. Syst. Signal Proc..

[9] Qiao Z., Lei Y., Lin J., Jia F. (2017). An adaptive unsaturated bistable stochastic resonance method and its application in mechanical fault diagnosis. Mech. Syst. Signal Proc..

[10] Feng Y., Lu B., Zhang D. (2117). Multiscale singular value manifold for rotating machinery fault diagnosis. J. Mech. Sci. Technol..

[11] Dragomiretskiy K., Zosso D. (2014). Variational mode decomposition. IEEE Trans. Signal Proc..

[12] Wang X.B., Yang Z.X., Yan X.A. (2018). Novel particle swarm optimization-based variational mode decomposition method for the fault diagnosis of complex rotating machinery. IEEE/ASME Trans. Mechatron..

[13] Yang Z.X., Zhong J.H. (2016). A hybrid EEMD-based SampEn and SVD for acoustic signal processing and fault diagnosis. Entropy.

[14] Smith J.S. (2005). The local mean decomposition and its application to EEG perception data. J. R. Soc. Int..

[15] Li Y., Liang X., Yang Y., Xu M., Huang W. (2017). Early Fault Diagnosis of Rotating Machinery by Combining Differential Rational Spline-Based LMD and K–L Divergence. IEEE Trans. Instrum. Meas..

[16] Wang L., Liu Z., Miao Q., Zhang X. (2018). Time–frequency analysis based on ensemble local mean decomposition and fast kurtogram for rotating machinery fault diagnosis. Mech. Syst. Signal Proc..

[17] Deng W., Yao R., Sun M., Zhao H., Luo Y., Dong C. (2017). Study on a novel fault diagnosis method based on integrating EMD, fuzzy entropy, improved PSO and SVM. J. Vibroeng..

[18] Lu C., Wang Z.Y., Qin W.L., Ma J. (2017). Fault diagnosis of rotary machinery components using a stacked denoising autoencoder-based health state identification. Signal Proc..

[19] Gao Y., Villecco F., Li M., Song W. (2017). Multi-Scale permutation entropy based on improved LMD and HMM for rolling bearing diagnosis. Entropy.

[20] Ling X., Yan X. (2014). Comparative study on the performance of LMD method and EMD method in steam turbine rotor fault diagnosis. J. Chin. Soc. Power Eng..

[21] Xu T., Yin Z., Cai D., Zheng D. (2017). Fault diagnosis for rotating machinery based on Local Mean Decomposition morphology filtering and Least Square Support Vector Machine. J. Intell. Fuzzy Syst..

[22] Rilling G., Flandrin P., Goncalves P. Empirical mode decomposition, fractional Gaussian noise and Hurst exponent estimation. Proceedings of the IEEE International Conference on Acoustics, Speech, and Signal Processing.

[23] Deering R., Kaiser J.F. The use of a masking signal to improve empirical mode decomposition. Proceedings of the IEEE International Conference on Acoustics, Speech, and Signal Processing.

[24] Nakayama J., Kanno K., Uchida A. (2016). Laser dynamical reservoir computing with consistency: An approach of a chaos mask signal. Opt. Express.

[25] Case Western Reserve University Case Western Reserve University Bearing Data Center. https://csegroups.case.edu/bearingdatacenter/pages/download-data-file.

